# Hybrid bilayer membranes on metallurgical polished aluminum

**DOI:** 10.1038/s41598-021-89150-2

**Published:** 2021-05-06

**Authors:** Tomas Sabirovas, Aušra Valiūnienė, Gintaras Valincius

**Affiliations:** 1 Institute of Chemistry, Faculty of Chemistry and Geosciences, Vilnius University, Naugarduko 24, 03225 Vilnius, Lithuania; 2 Institute of Biochemistry, Life Sciences Center, Vilnius University, Sauletekio ave. 7, 10257 Vilnius, Lithuania

**Keywords:** Biochemistry, Chemistry

## Abstract

In this work we describe the functionalization of metallurgically polished aluminum surfaces yielding biomimetic electrodes suitable for probing protein/phospholipid interactions. The functionalization involves two simple steps: silanization of the aluminum and subsequent fusion of multilamellar vesicles which leads to the formation of a hybrid bilayer lipid membrane (hBLM). The vesicle fusion was followed in real-time by fast Fourier transform electrochemical impedance spectroscopy (FFT EIS). The impedance-derived complex capacitance of the hBLMs was approximately 0.61 µF cm^−2^, a value typical for intact phospholipid bilayers. We found that the hBLMs can be readily disrupted if exposed to > 400 nM solutions of the pore-forming peptide melittin. However, the presence of cholesterol at 40% (mol) in hBLMs exhibited an inhibitory effect on the membrane-damaging capacity of the peptide. The melittin-membrane interaction was concentration dependent decreasing with concentration. The hBLMs on Al surface can be regenerated multiple times, retaining their dielectric and functional properties essentially intact.

## Introduction

Artificial lipid membranes provide a suitable platform for investigations of biological membrane related processes such as membrane-protein interactions^1,2^, ion permeability^3–5^, redox reactions^6,7^, etc. Medical diagnostics^8–10^, drug screening^11,12^, and environmental controls^13–15^ are often based on artificial lipid membranes by the combination of biological recognition with a physicochemical transducer. The obvious advantages of membrane-based biosensors are the nature-like environment and the relatively simple preparation through a self-assembly process.

Emanating from the pioneering work of Mueller on bilayer lipid membranes (“black lipid membranes”)^16,17^, various approaches were developed for the formation of artificial lipid membranes. Among these, solid supported lipid bilayers received considerable recognition. Several different strategies can be adopted for the formation of the lipid membrane on a solid support. One of them is supported lipid bilayers, where lipid bilayer is formed directly on solid support such as oxidized silicon^18^, silica^19^, mica^20^, aluminum^21^, titanium^22^, etc. The advantage of using solid supports is an increase in the robustness and stability of the artificial membranes as well as the ability to probe the surface with powerful analytical techniques (e.g. quartz crystal microbalance^23^, atomic force microscopy^24^, surface plasmon resonance^25^, electrochemical impedance spectroscopy^26^). The major drawback of supported lipid bilayers is the thinness of the water layer between the bilayer and solid support, which can present unfavorable interactions to a) the incorporation of transmembrane proteins, b) protein-substrate interactions, and c) loss of protein lateral mobility^27^.

Another widely used strategy is to form artificial bilayers on modified surfaces with a polymer layer or self-assembled monolayer^28–35^. Such functionalization of the surface decouples the artificial membrane from the substrate and, in principle, more readily allows for the transmembrane proteins to preserve their function. Modification of the surface with molecular anchors can be easily achieved either by thiol-metal or silane-oxide surface chemistry. Gold^34^ as a substrate has been most extensively studied for the formation of bilayer lipid membranes but other surfaces are viable choice as well e.g. silica^36^, indium tin oxide (ITO)^37^, fluorine-doped tin oxide (FTO)^38^, cadmium tin oxide (CTO)^39^, Ti/TiO_2_^40–42^, aluminum^43–46^, etc. In the majority of these cases, such surfaces require special preparation techniques e.g. magnetron sputtering, which increases the total expense towards investigations of membrane related processes or for applications of electrochemical/electroanalytical devices. Therefore, the magnitude of the literature clearly indicates metallurgical surfaces have extensive potential to be used as membrane-based biosensor platforms. Previously, we investigated metallurgical titanium surface for the phospholipid bilayer formation^41,42^, since titanium exhibits excellent biocompatibility, high chemical stability, and non-toxicity^47^. Nevertheless, preparation of metallurgical titanium surface e.g. surface polishing is challenging due to the hardness of material. Therefore, other cost-efficient and soft metal surfaces should be explored for phospholipid bilayer formation as well. One of them is aluminum. The major advantages of aluminum compared to titanium surface is the near effortless preparation of the surface due to the softness of the material and its higher affinity towards silane-based compounds^48^. The later has been already exploited to make aluminum surfaces corrosion resistant or to display self-cleaning surface properties^49,50^. Due to the easily achievable structuration of cylindrical pores by an anodization process, aluminum surfaces found usage for pore-spanning bilayers^45,46,51^. Also, metallic nanostructured aluminum surfaces is an emerging tool for SPR based biosensing applications^52–55^, since aluminum generates surface plasmons in the visible and UV regions^56^. Moreover, evidence exists that Al substrates can be used for surface-enhanced fluorescence and surface-enhanced Raman spectroscopies^57–59^. The challenge of surface oxidation and material degradation (corrosion and pitting) have to be faced. These issues are usually addressed by the means of oxygen plasma to produce an oxide protecting layer on the aluminum surfaces. We believe, protective self-assembled silane monolayers and phospholipid overlayers could potentially be used for substrate protection in biologically relevant environments and at the same time expand the field of the sensitive optical biosensor applications based on metallurgical aluminum surface.

The objective of this work was to design biomimetic hybrid bilayer lipid membranes (hBLMs) on the surface of metallurgical polished aluminum. Biomimetic hBLMs on Al surfaces may be of use in biomedical applications both as membrane-based bioanalytical devices or functional interfaces capable of immobilization of membrane bound proteins and peptides. In this study we developed a simple procedure for the formation of the hybrid bilayers and investigated the feasibility of their repetitive regeneration without losing functional properties. Using the membrane disrupting peptide, melittin, we showed that such hBLMs can be effectively used for the detection of membrane damaging agents such as pore forming peptides and proteins.

## Experimental

### Preparation of the metallurgical aluminum surface

Metallurgical aluminum plate (25 × 55 mm, thickness 0.125 mm) (> 99.0%, Goodfellow GmbH) was polished with a 200,00 grit diamond lapidary paste with a diamond powder (size ≤ 0.1 µm) concentration of 50% at the speed of 1000 rpm until a mirror-like surface was observed. The process of surface polishing was carried out in the air. The polished aluminum plate was then cleaned by ultrasound for 10 min in (i) hexane (≥ 99%, Reachem, Slovakia), (ii) 2-propanol (≥ 99.5%, Sigma-Aldrich), (iii) 2 min in Milli-Q water (Milli Q-plus-Millipore system (USA)), then dried under a nitrogen gas stream.

### Self-assembled monolayer formation

Freshly polished and cleaned metallurgical aluminum surfaces, as just described above, were used for the formation of octadecyltrichlorosilane (OTS) (> 90%, Sigma-Aldrich) self-assembled monolayers. The silanization solution was prepared by heating 45 mL heptane to 60–65 °C and adding OTS at 2.5 mM concentration. The aluminum plate was immersed into a silanization solution for 45 min, then heated at 100 °C for 1 h in air to remove adsorbed water and solvent residues.

### Hybrid bilayer lipid membrane (hBLM) completion

Hybrid bilayer lipid membranes were formed by the vesicle fusion method described in the reference^60^. Briefly, vesicle solutions were prepared from 1,2-dioleoyl-sn-glycero-3-phosphocholine (DOPC) (Avanti Polar Lipids, Inc., USA) or molar ratio mixtures of 60% DOPC and 40% cholesterol (Chol) (Avanti Polar Lipids, Inc., USA) in chloroform (99%, Sigma-Aldrich) to a concentration of 10 mM. A desired amount of the lipid solutions was transferred to separate vials and evaporated under a nitrogen stream until the formation of the lipid film was observed. The lipid film was re-suspended in phosphate buffer (0.1 M NaCl, 0.01 M NaH_2_PO_4_, pH adjusted to 7.1 with NaOH), to a final 1.5 mM total lipid concentration. The NaCl, NaH_2_PO_4_ and NaOH were purchased from Reachem Slovakia p. a.

### Regeneration of self-assembled monolayer

Regeneration of the self-assembled monolayer was carried as following: (i) hybrid bilayer lipid membranes were formed on silanized metallurgical Al surfaces via the vesicle fusion method, (ii) the cell was washed with a copious amount of phosphate buffer (pH 7.1), (iii) the phosphate buffer solution was drained out of the cell, (iv) a solution of 2-propanol in Milli-Q water (volume % ratio 50/50) was added into the cell with a pipette and vigorously agitated by refluxing (partial withdrawal and re-adding using a pipette) the 2-propanol/H_2_O solution in contact with the surface a few times to disintegrate the hybrid bilayer (the action was repeated for 5 times), (v) the cell was lastly washed with a copious amount of phosphate buffer (pH 7.1) and thus completing the first regeneration.

### Electrochemical measurements

Measurements of electrochemical impedance spectroscopy (EIS) and cyclic voltammetry (CV) were carried out in phosphate buffer (pH 7.1). A three-electrode conventional system was used for measurements configuration, where the aluminum plate served as the working electrode, saturated silver-silver chloride (Ag/AgCl/NaCl_(sat.)_) microelectrode (M-401F, Bedford, USA) as a reference electrode and platinum (99.99% purity, Aldrich) wire as an auxiliary electrode, which was coiled around the barrel of the reference electrode. CV measurements were carried out at a scan rate of 10 mV s^-1^ and a scan step at 1 mV using the µAutolab (Utrecht, the Netherlands). The potential was scanned between − 1.1 V and − 0.5 V versus Ag/AgCl/NaCl_(sat.)_.

EIS were measured using µAutolab (unless indicated otherwise) (Utrecht, the Netherlands) in a frequency range from 0.1 Hz to 50 kHz. In cases where it was deemed necessary to avoid the influence of corrosion of the aluminum surface and to investigate the kinetics of the vesicle fusion process, fast Fourier transform (FFT) electrochemical impedance spectrometer EIS-128/16 (University of Kiel, Germany)^61^, which registers EI spectrum very fast (within 1.06 s in a frequency range between 1.5 Hz and 50 kHz), was used instead.

An additional platinum wire electrode (connected to the 1 μF capacitor) was used as a quasi-reference electrode to reduce the impedance of the reference electrode at the higher frequencies for FFT-EIS measurements. The obtained data are normalized to the geometric surface area of 0.32 cm^2^ of the working electrode. The electrode potentials in the article are versus Ag/AgCl/NaCl_(sat.)_.

All measurements were performed at room temperature of 21 ± 3 °C. Multiple experiments (2 to 15) were carried out for determining standard deviations.

### Attenuated total reflection Fourier transform infrared (ATR-FTIR) spectroscopy

Fourier-transform infrared (FTIR) spectra of the aluminum functionalized with OTS self-assembled monolayer were registered using the spectrometer Bruker Alpha (Germany) equipped with a diamond attenuated total reflection (ATR) detector. The spectra were acquired in the wavenumbers range from 4000 cm^−1^ to 400 cm^−1^ at 4 cm^−1^ resolution from 100 scans. The spectrum of the aluminum was used as a reference and all experiments were carried out at ambient conditions.

### Contact angle measurements

The contact angle was measured with Theta Lite Optical Tensiometer from Biolin Scientific (Finland) company by placing 6 droplets of 10 µL Milli-Q water. The contact angle measurements were performed immediately after the aluminum plate was cleaned or silanized.

## Results and discussion

### Formation of self-assembled monolayer

The formation of the artificial membranes on self-assembled monolayers (SAM) requires sufficient surface free energy^62^. For that reason, the aluminum surface was functionalized with octadecyltrichlorosilane (OTS) monolayer by a simple silanization procedure. The most straightforward way to detect self-assembled monolayer (SAM) is to measure wetting properties–contact angles (CA) before and after silanization of the Al surface. Metallurgical aluminum surfaces exhibited hydrophilic properties showing CA values of 41.72° ± 3.96°. After the silanization procedure, the values of contact angles increased to 105.13° ± 2.02° clearly demonstrating hydrophobic properties of the silanized Al surface. The obtained contact angle values of 105.13° ± 2.02° for the silanized Al surface are slightly higher than those obtained on OTS monolayer formed on mechanically polished Ti surface (102.27° ± 1.76°)^41^ and lower than those obtained on OTS formed on FTO (119° ± 7°)^38^ surface. However, the change of the wetting characteristics from hydrophilic to hydrophobic in all cases attests for the formation of the organic OTS monolayer on the surface, and consequently shows that functionalized metallurgical aluminum surface has sufficient surface free energy, needed for further immobilization of phospholipid layer^62^.

**Figure 1:**
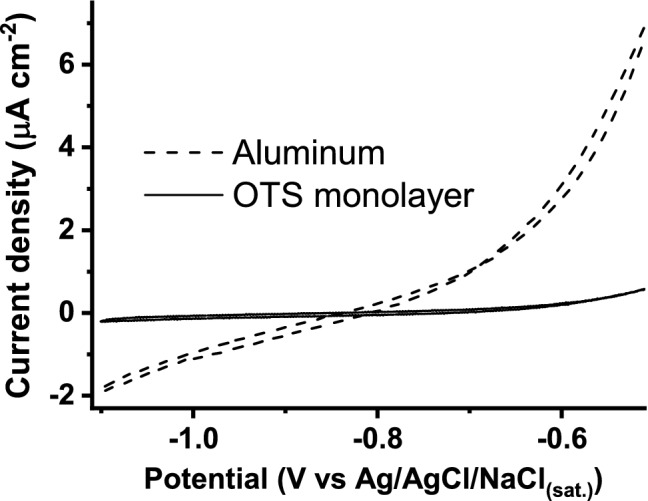
Cyclic voltammograms of the metallurgical aluminum surface before and after silanization at the scan rate of 10 mV s^−1^ in phosphate buffer, pH 7.1.

Metallurgical Al surfaces before and after silanization procedure were tested with cyclic voltammetry (CV) in phosphate buffer solution (pH 7.1) in order to determine the potential range of non-Faradaic processes needed for further surface evaluation with EIS. The potential range for the CV measurements was chosen empirically by measuring the equilibrium potential, which was around − 0.65 V versus Ag/AgCl/NaCl_(sat.)_, and increasing the potential to more negative and positive values. Cyclic voltammetry curves showed that Faradaic processes occur on the Al surface over the potential range from − 1.1 V to − 0.5 V (Fig. 1, dashed line). Usually, it is expected that aluminum is covered with a naturally occurring Al oxide which at neutral pH (~ 7) is expected to be electrochemically stable^63^. However, in a presence of the chloride anions the passive oxide film becomes unstable and local corrosion occurs^64^. Therefore, ideal polarizability was not observed on the aluminum surface (Fig. 1, dashed line), since oxidation and reduction processes take place in the investigated potential range. After the formation of OTS monolayer on the Al surface, even in the presence of chloride anions, almost ideal polarizability was observed in the potential range from − 1.1 V to − 0.65 V (Fig. 1, solid line). This effect can be attributed to the formation of a self-assembled monolayer resulting in the blockage of the interface aluminum–buffer solution.

**Figure 2:**
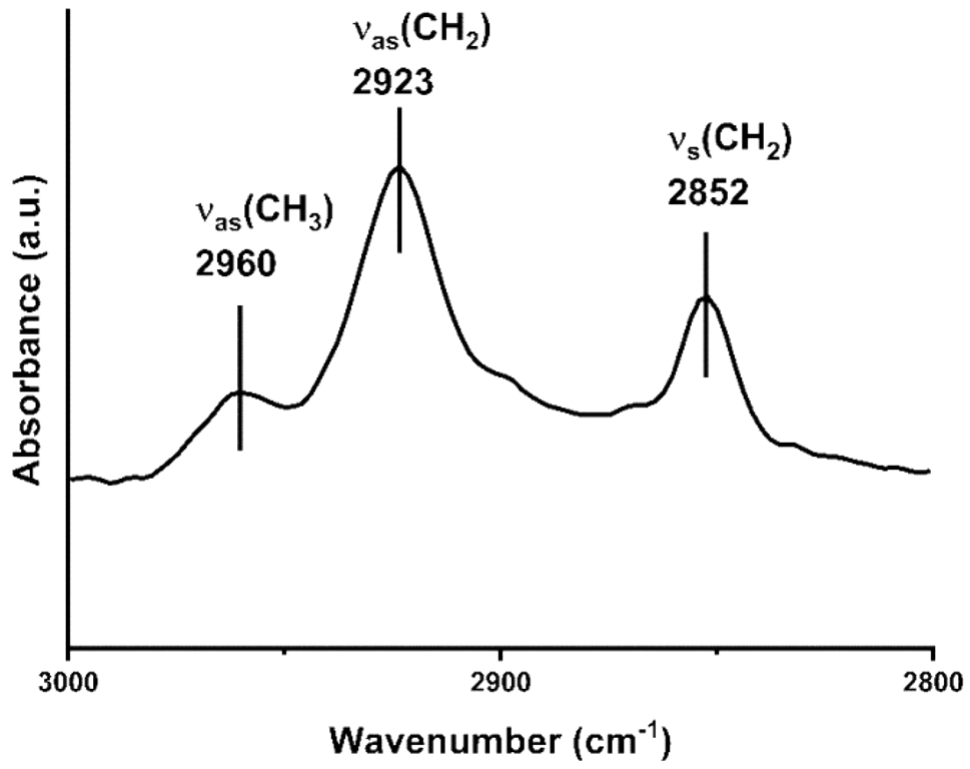
C–H stretching region of ATR-FTIR spectra of OTS self-assembled monolayer formed on the metallurgical polished aluminum surface.

The OTS monolayers on the Al surface were also analyzed with ATR-FTIR spectroscopy, which allows assessing the order in alkyl chains of the SAM^65,66^. Figure 2 displays the C-H stretching region where three vibration absorbance bands are observed. The bands at 2852 cm^−1^ and 2923 cm^−1^ can be assigned to C-H symmetric (ѵ_s_(CH_2_)) and asymmetric (ѵ_as_(CH_2_)) stretching of methylene, respectively. The weak band at 2960 cm^−1^ is assigned to methyl asymmetric stretching (ѵ_as_(CH_3_))^65,66^. The position of ѵ_as_(CH_2_) band can be used for the evaluation of the order in alkyl chains^66^. For highly ordered octadecanethiol SAMs on the gold surface, the band of ѵ_as_(CH_2_) appears at 2917 cm^−1^, shifting to higher wavenumbers as the gauche conformations along with the methylene chain increases (i.e. disorder)^65,66^. In our case, ѵ_as_(CH_2_) appears at 2923 cm^−1^ indicating some disorder in the OTS SAMs. Also, the lower band intensity of ѵ_s_(CH_3_) relative to ѵ_s_(CH_2_) shows that the methyl groups are parallel to the surface, as observed in mixed SAMs with lower packing densities on gold^67^.

### Formation of hybrid bilayer membranes

**Figure 3:**
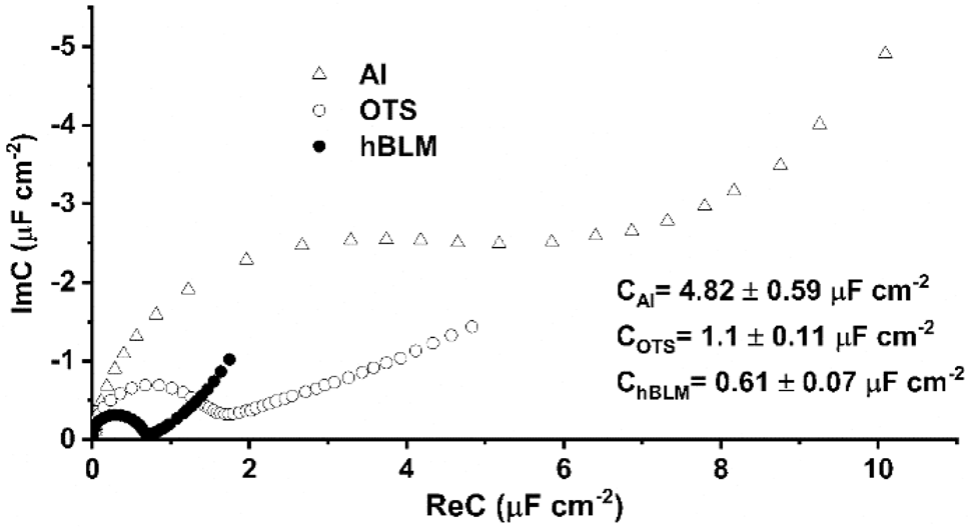
Electrochemical impedance spectra in Cole–Cole plot of Al, OTS and DOPC:Chol (molar % ratio 6:4) hBLM, formed by vesicle fusion method after 30 min incubation time. The applied potential: − 0.7 V versus Ag/AgCl/NaCl_(sat.)_. Mean complex capacitance values with standard deviations are displayed as well.

**Figure 4:**
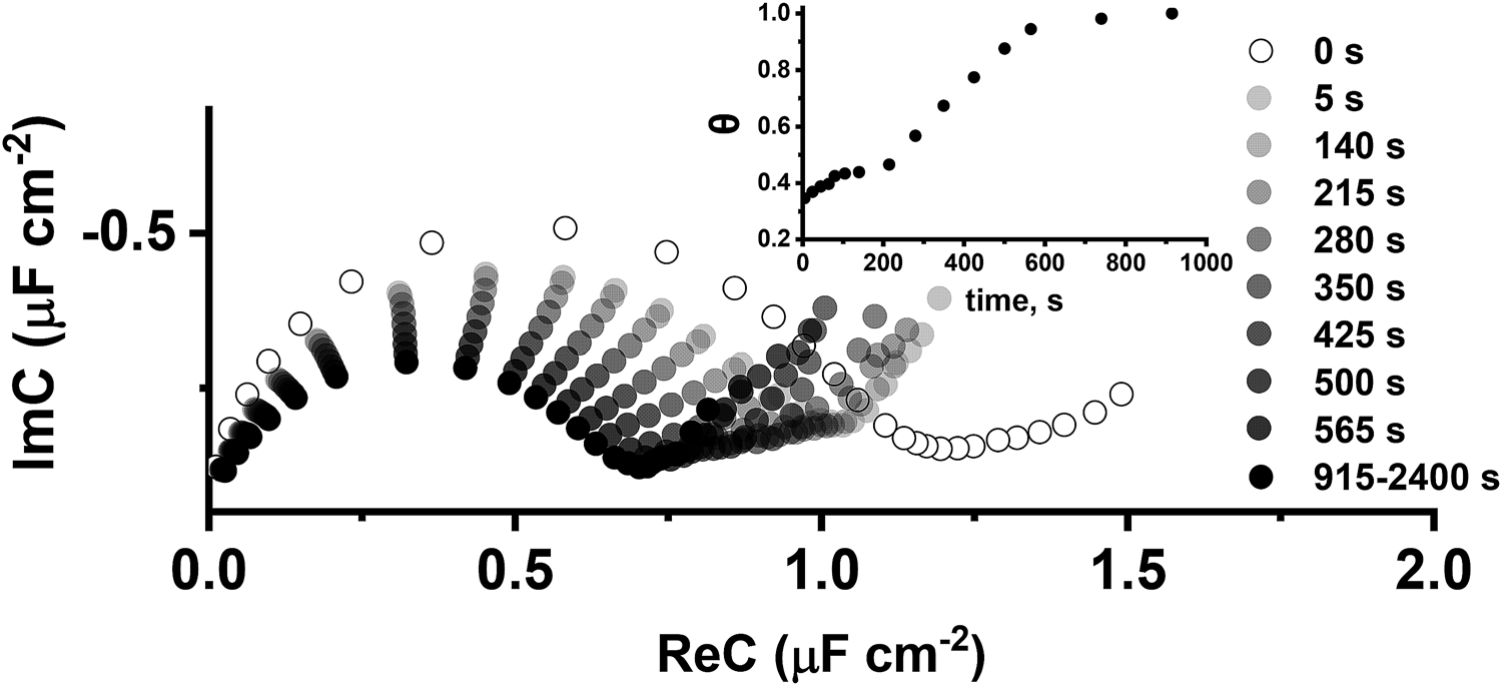
Cole–Cole plots of FFT impedance spectra of the DOPC:Chol (molar % ratio 6:4) vesicle fusion process on silanized aluminum surface. The applied potential: **− **0.7 V versus Ag/AgCl/NaCl_(sat.)_. Inset—surface coverage, θ, of the lipid layer over time.

**Figure 5:**
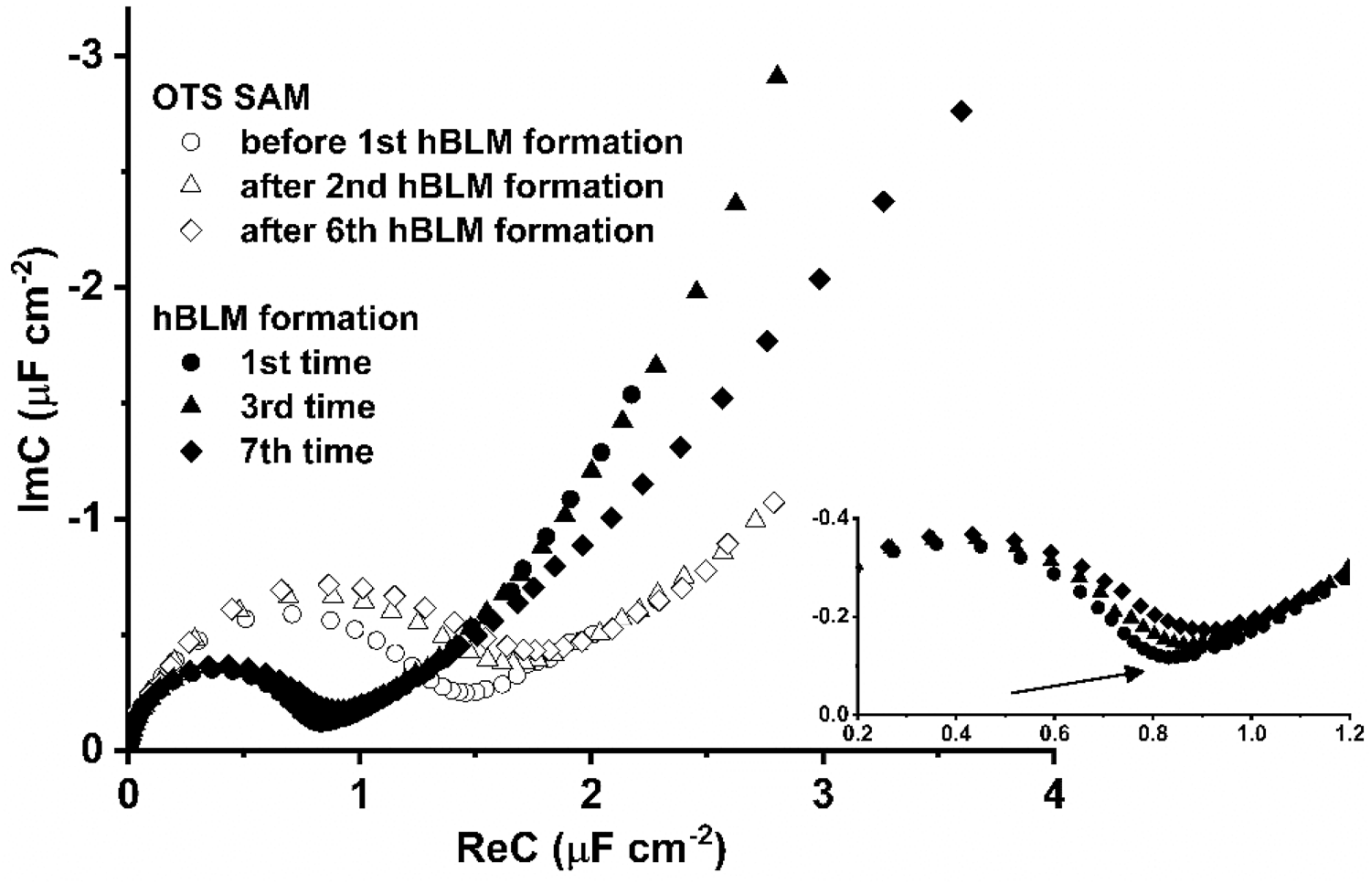
Cole–Cole plots of electrochemical impedance spectroscopy of the OTS SAM regeneration for DOPC:Chol (molar % ratio 6:4) hybrid bilayer formation on the metallurgical aluminum surface. The applied potential for EI measurements: − 0.7 V versus Ag/AgCl/NaCl_(sat.)_. Inset shows an enlarged part of the complex capacitance plot of the hBLM formations.

Contact angle, cyclic voltammetry, and ATR-FTIR measurements (Figs. 1, 2) attest for the formation of OTS monolayers needed for immobilization of the hybrid bilayer lipid membranes. EIS, which is capable of detecting the dielectric properties of the surface, was applied for investigation of the hybrid bilayer formation on the metallurgical aluminum surface. Typically, the semi-circular shape of EI response in the complex capacitance plot is observed for ideally polarizable interfaces demonstrating nearly ideal capacitive behavior^26,34,41,68^. The approximate complex capacitance values can be obtained from the Cole–Cole plots by taking the radius of the semi-circle and multiplying it by two. For detailed capacitance values and equivalent circuits used to model electrochemical impedance data please see Supporting material data (Fig. 1S-5S, Table 1S-4S).

Figure 3 shows electrochemical impedance spectra in Cole–Cole plots of the metallurgical Al surface functionalized with an OTS monolayer and a hBLM. Initially, the metallurgical aluminum surface exhibited relatively high values of complex capacitance reaching 4.82 ± 0.59 µF cm^−2^ (Fig. 3, open triangles). As expected, the silanization of the aluminum surface causes the “shrinkage” of the semi-circle demonstrating a decrease of complex capacitance by approximately four-fold to 1.1 ± 0.11 µF cm^−2^ (Fig. 3, open circles) and indicating the formation of the dielectric layer of OTS monolayer on the Al surface. By comparison, the OTS analog octadecanethiol, which forms ordered, compact SAMs on gold surfaces^34,69^ exhibit complex capacitance values well below 1 µF cm^−2^. The difference in the capacitance values is an indication of void spaces (defects) in the SAM exposing the Al surface and, consequently, these defects increase the complex capacitance of the SAM^70^. Taking into account that OTS silanized mechanically polished titanium^41^ and magnetron sputtered titanium^40^ surfaces were successfully applied for the formation of hBLMs albeit exhibiting complex capacitance values above 1 µF cm^−2^, the silanized metallurgical Al surface was also tested for the formation of bilayer lipid membranes via vesicle fusion. After immobilization of hBLMs, the complex capacitances decreased to 0.61 µF cm^−2^ ± 0.07 µF cm^−2^ (Fig. 3, filled circles), which is a value typical for phospholipid bilayers.

The process of vesicle fusion was monitored in real-time using FFT-EIS method by measuring EIS every 5 s for 40 min. Figure 4 shows the complex capacitance evolution after injection of DOPC:Chol (molar % ratio 6:4) vesicles on the OTS monolayer. The decrease of the complex capacitance was noticeable after 5 s indicating that vesicle fusion process was triggered instantaneously (Fig. 4). As incubation time increases, the semicircle in Fig. 4 “shrinks” further indicating the decrease of the complex capacitance from 0.86 µF cm^−2^ (after 5 s) to 0.61 ± 0.07 µF cm^−2^. The completion of vesicle fusion typically occurred within approximately 500 s. Fusion completion can be detected by the limit capacitance value of 0.61 ± 0.07 µF cm^−2^ which is comparable to those obtained for bilayer lipid membranes formed on gold (~ 0.6 μF cm^−2^)^33,34^, FTO (0.82 ± 0.10 μF cm^−2^)^38^ and mechanically polished Ti surfaces (0.61 µF ± 0.06 µF cm^−2^ )^41^.

The vesicle fusion progression can be followed by the parameter, θ, which approximately indicates the fraction of the surface covered by the hBLM:

1 $$ \theta = \frac{{C_{SAM} - C_{meas} }}{{C_{SAM} - C_{hBLM} }} $$

where C_SAM_—the magnitude of complex capacitance of self-assembled monolayer, C_meas_—the magnitude of the complex capacitance measured at a specific time during the vesicle fusion and C_hBLM_—the magnitude of complex capacitance at 100% coverage by the hybrid bilayer membrane (0.61 µF cm^−2^).

As observed in Fig. 4 inset, the step-like increase of surface coverage to θ = 0.34 was observed within the first 5 s. At 100 s the coverage reached its first plateau at θ = 0.42. The increase resumed at approx. 180 s reaching coverage of θ = 0.94 in 560 s. Then, slow convergence towards θ → 1 was observed over the next 350 s. The formation of a 100% hBLM was typically observed between 900 and 1000 s. For the sake of reproducibility, we typically use 30 min (1800s) of vesicle fusion during which almost defect-free hBLMs were formed.

The trace shown in Fig. 4 inset represents a typical kinetics curve for amphiphilic molecules undergoing two-stage adsorption. During the first stage molecules adsorb onto the surface of adsorbate (in our case the SAM) establishing maximal area of contact. In our case this corresponds to a horizontal orientation (striped phase) of the phospholipid molecules on anchor SAMs. Then, as adsorption proceeds, the molecules rearrange themselves into a more compact arrangement corresponding, in our case, to a phospholipid monolayer oriented nearly parallel to the surface normal.

In conclusion, our finding allows us to conclude that the vesicle fusion led to a formation of intact, essentially defect free hBLMs. This leads to a broader conclusion of applicability of the metallurgical Al surface for the formation of hybrid bilayer lipid membrane (hBLM) by the vesicle fusion method.

### Regeneration of self-assembled monolayer for the formation of the artificial membrane

Regeneration experiments were carried out to explore the possible exploitation of the OTS SAMs for multiple hBLM formations. Firstly, the OTS SAM was monitored with EIS after the silanization procedure. After that, the hBLM was formed via the vesicle fusion method, and the formation of sequential hBLMs was monitored with EIS after each reconstitution. Figure 5 displays EIS data obtained after 7 regenerations (details for regeneration procedure are presented in Experimental. It was observed that complex capacitance values of the SAM and hBLMs slightly changed after each reformation of an hBLM. Initially, the OTS complex capacitances were 1.16 µF cm^−2^ (Fig. 5, open circles). Over the course of the regeneration sequence the values of the complex capacitance of the SAM increased to 1.42 µF cm^−2^ after the 6th hBLM formation (Fig. 5, open diamonds). This feature can be attributed to the loss of the dielectric layer and adsorption of water molecules into the SAM. The sequential hBLMs follow the same tendency of an increase in the complex capacitance value, however, this tendency was less noticeable. Particularly, the complex capacitance values increased slightly from 0.68 µF cm^−2^ following the first formation of hBLM to 0.74 µF cm^−2^ of the 7th formation of hBLM (Fig. 5, filled diamonds). The apparent transformation of EIS occurs at the end of the semi-circle as indicated by the arrow in Fig. 5, inset. The end of the semi-circle consistently shifts towards the “northeast” direction with increasing numbers of SAM regenerations. Such EIS responses are usually obtained when the size of the defects in the membrane increases^26^. Significantly, however, even though sequential, multiple formation of hBLMs on regenerated SAMs increases SAM defectiveness, these OTS SAMs on metallurgical aluminum surfaces can be regenerated up to 6 times and re-used for multiple formations of hBLMs.

### Melittin interaction with artificial membrane

**Figure 6:**
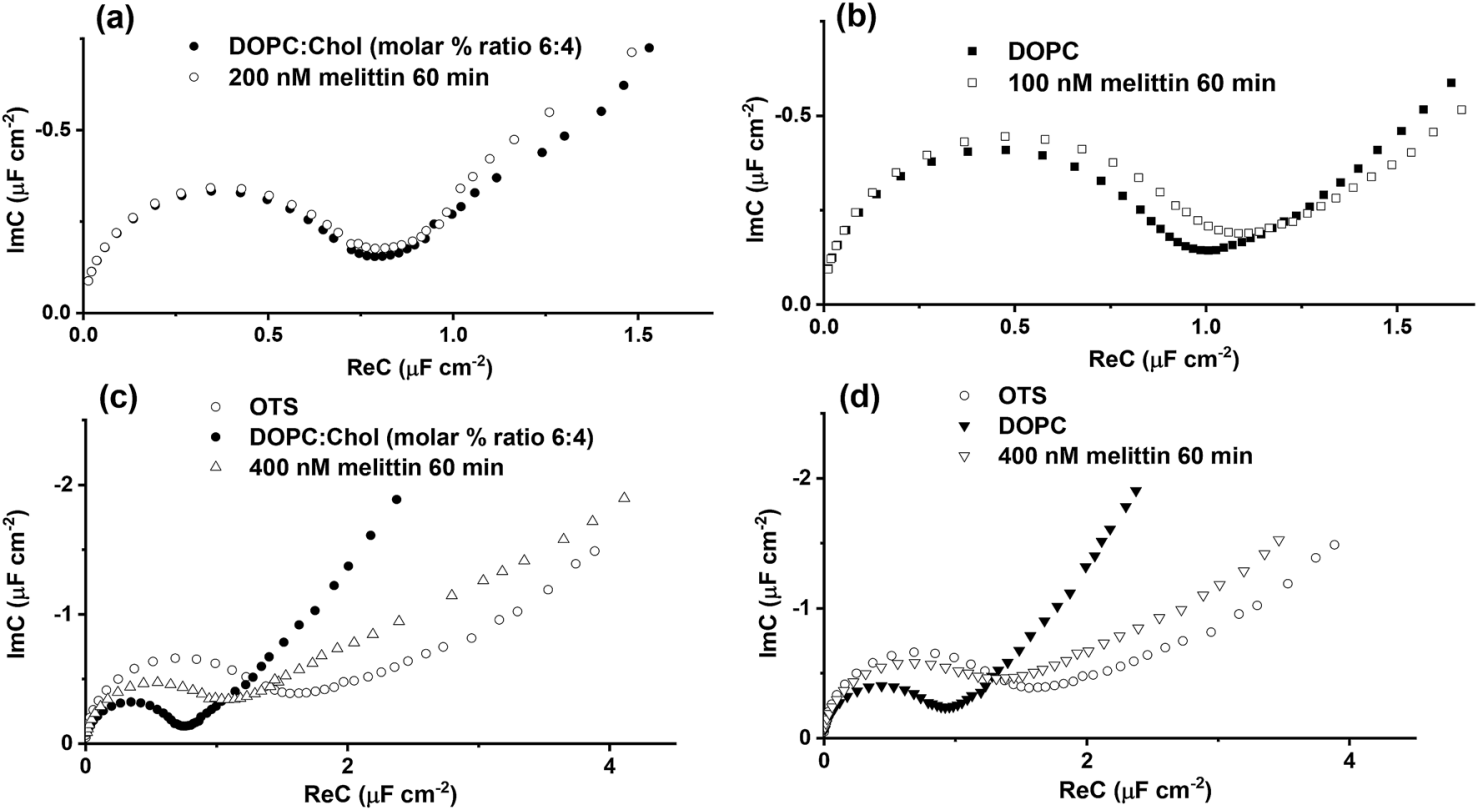
Electrochemical impedance spectra in Cole–Cole plot of melittin interaction: (**a**) and (**c**)—with DOPC:Chol (molar % ratio 6:4), (**b**) and (**d**)—with DOPC hybrid bilayer lipid membranes. The applied potential: − 0.7 V versus Ag/AgCl/NaCl_(sat.)_.

Melittin, a major component of bee venom^71^, is a 26 amino acid cationic peptide existing as a monomer in an aqueous environment^72,73^. Its amphipathic properties allow it to easily associate with the structures like lipid bilayers. When bound to lipid bilayers melittin adopts an α-helical conformation and disrupts the bilayer lipid membrane by the formation of a pore. Figure 6 displays Cole–Cole plots of the hBLMs after a 60 min interaction with solutions containing various amounts of melittin. In all cases exposure of the hBLM to melittin triggers EIS spectral changes consistent with the disruption of the phospholipid insulating layer and increases of a complex capacitance. The effect of melittin was found to be dependent on concentration and lipid composition. After exposure of DOPC:Chol (molar % ratio 6:4) hBLM to 200 nM melittin solution, the EI spectra (Fig. 6a) remained almost unchanged. An increase of melittin concentration to 400 nM results in a significant increase of the complex capacitance of the DOPC:Chol hBLM from 0.64 µF cm^−2^ to 0.94 µF cm^−2^ (Fig. 6c). Further increase in melittin concentration to 500 nM results in a disintegration of the hBLM which is evident from the complex capacitance increase to 1.06 µF cm^−2^ (data not shown), a value close to the complex capacitance value of the bare anchor SAM with no phospholipid overlayer.

In cholesterol-free hBLMs (100% DOPC) the melittin effect on membrane capacitance is quite significant even at low melittin concentrations. At 100 nM, the complex capacitance of the DOPC bilayer slightly increased from 0.80 µF cm^−2^ to 0.88 µF cm^−2^ (Fig. 6b). However, at 400 nM of melittin the cholesterol-free DOPC hBLM was fully disrupted as indicated by a large complex capacitance increase from 0.80 µF cm^−2^ to 1.16 µF cm^−2^ (Fig. 6d).

Our findings indicate an inhibitory effect by cholesterol towards melittin induced damage of the bilayer^74,75^. Actually, the membrane-bound melittin can exist in two orientations: parallel, where only interactions with lipid head group occurs, and perpendicular, where it reconstitutes into the hydrophobic core of the membrane, leading to pore formation^76^. These orientations are highly dependent on melittin concentration. At lower concentrations (approximately below 0.5 µM^77^, however it must be noted that exact concentration depends on the many factors e.g. lipids, ionic strength, pH)^78–80^, the peptide binds to the surface of the bilayer in parallel conformation. In our case the major difference between cholesterol-free and cholesterol-loaded hBLMs is observed at low melittin concentrations. We speculate that the melittin peptide cannot penetrate the membranes with cholesterol and remains oriented parallel but at concentrations, above 400 nM, the peptide overcomes this resistance to penetration and adopts more vertical orientations, creating pores (defects), and increasing the capacitance complex of the system.

The disruptive effect of the peptide on the hybrid bilayers can be utilized to design impedimetric biosensors for the detection of membrane damaging toxins such as melittin. For this purpose, the extent of damage can be quantified as follows:

2 $$ MD = 1 - \left( {\frac{{C_{SAM} - C_{melittin} }}{{C_{SAM} - C_{hBLM} }} } \right) $$

where MD denotes the membrane damage: C_SAM_—is the complex capacitance value of the OTS monolayer, C_melittin_—the complex capacitance of the hBLM after 60 min of exposure to melittin, C_hBLM_—the complex capacitance value of the hBLM.

**Figure 7:**
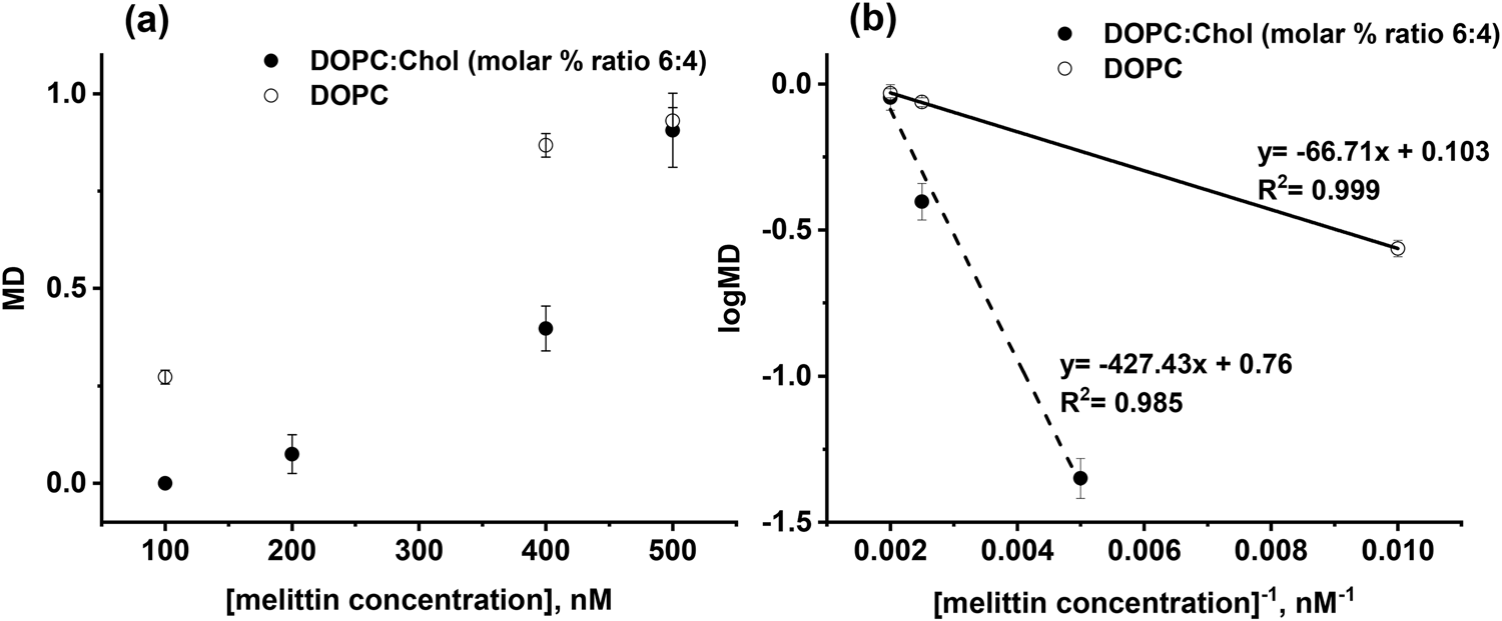
(**a**)—Dependence of the membrane damage, MD, on melittin concentration, (**b**)—same data depicted in logMD versus inverse of melittin concentration.

Figure 7a displays the dependence of MD versus melittin concentration. The curve indicates a non-linear dependence. Therefore, for analytical purposes, the semilogarithmic inverse concentration plot (Fig. 7b) is better, which we found empirically, and exhibits relatively a good linear dependence in the concentration range from 100 to 500 nM. We believe this interval of melittin concentration can be extended by properly adjusting phospholipid composition of hBLMs which requires further investigation.

### Conclusions

In this work we assembled hybrid bilayer lipid membranes on polished metallurgical aluminum surfaces. Functionalized by an OTS monolayer the Al surface triggers fast and effective vesicle fusion leading to a formation of a layer of phospholipid. Experimental data suggest that 900 s are sufficient for the formation of compact and highly insulating hBLMs with the complex capacitance value of 0.61 ± 0.07 µF cm^−2^ as measured by the electrochemical impedance spectroscopy. The hBLMs can be regenerated multiple times, which makes hBLMs on metallurgical polished Al a convenient reusable platform for peptide membrane interaction studies.

The hBLMs in our study are sufficiently fluid to allow a biologically relevant response to the membrane damaging agent, melittin from bee venom. The response was found to be dependent on cholesterol content in the hBLMs, with cholesterol acting as a moderate inhibitor of melittin damage, consistent with earlier studies on vesicles^74,75,81^. The melittin effect was reproducible and concentration dependent which suggests utility of the hBLMs on metallurgical Al as a robust platform for biosensor design.

Our study also shows that the regenerable hBLMs on Al are superior to the ones on gold substrates not only because of the price considerations, but also because of the possibility of the multiple usage and regeneration without considerable loss of the integrity as is typically the case for the gold-tethered BLMs^82^. Commercial availability of low-priced metallurgical Al surfaces, straightforward surface preparation and surface regeneration by a simple mechanical polishing can provide a superior and a convenient platform for cost-efficient membrane-based bioanalytical devices.

## Supplementary Information

Supplementary Information
