# Supplementary material for: Hybrid bilayer membranes on metallurgical polished aluminum
Source: Sci Rep. 2021 May 6;11:9648. doi: 10.1038/s41598-021-89150-2 (PMC8102548; doi:10.1038/s41598-021-89150-2)
Supplement: Supplementary file 1 — Supplementary Information [file 41598_2021_89150_MOESM1_ESM.docx]

Supplementary Material

**Hybrid bilayer membranes on metallurgical polished aluminum**

Tomas Sabirovas^2^, Aušra Valiūnienė ^⁎1^, Gintaras Valincius^2^

^1^ Vilnius University, Faculty of Chemistry and Geosciences, Institute of Chemistry, Naugarduko 24, Vilnius, LT-03225, Lithuania

^2^ Vilnius University, Institute of Biochemistry, Life Sciences Center, Sauletekio ave. 7, Vilnius, LT-10257, Lithuania

^⁎^ Corresponding author. E-mail: [ausra.valiuniene@chf.vu.lt](about:blank)

1. **EIS data modeled to an equivalent circuit**

Figure 1S displays the equivalent circuits used in the modeling of electrochemical impedance spectra. Model A was to fit self-assembled monolayers and model B: hybrid bilayer lipid membranes. The frequency range for fitting EIS data was chosen between 50 kHz and 100 Hz to fit the semi-circle shape of EIS data in Cole-Cole plots.

|   **A** |   **B** |
| --- | --- |

**Figure 1S.** Equivalent circuits used in the modeling of electrochemical impedance spectra. Model A was used to model OTS monolayer; model B: to model hybrid bilayer lipid membranes [^[[1]](#endnote-1)^]. R_s_ is the solution resistance, CPE is constant-phase element coefficient and R_m_ is membrane resistance.

Figures 2S, 3S, 4S, 5S displays EIS data in the work with the solid lines representing fits to the data. The EIS curves were fitted either to model A or model B (Figure 1S.) depending on the functionalized Al surface: either self-assembled monolayer or hybrid bilayer lipid membrane, respectively. The fitting parameters for the data shown in figures are displayed in the tables (table 1S, 2S, 3S, 4S) below the figures. R_s_ represents the solution resistance, CPE represents constant-phase element coefficient, α_CPE_ represents the exponent of the constant phase element, R_m_ represents membrane resistance and parameter χ^2^ represents an objective function value of the best fit.

**Figure 2S.** Electrochemical impedance spectra in Cole-Cole plot of OTS and DOPC:Chol (molar % ratio 6:4) hBLM. The applied potential: -0.7 V vs Ag/AgCl/NaCl_(sat.)_. The solid lines represent fits to the data

**Table 1S.** EIS fitting farameters for the data shown in figures 2S

| Surface | R_s_, Ω·cm^2^ | CPE, (µF cm^−2^ s^(α−1)^) | α_CPE_ | R_m_, kΩ·cm^2^ | χ^2^(×10^5^) |
| --- | --- | --- | --- | --- | --- |
| OTS monolayer | 41.5 ± 0.19 | 2.78 ± 0.05 | 0.93 | — | 0.62 |
| hBLM | 41.56 ± 0.08 | 1.06 ± 0.03 | 0.94 | 31.99 ± 1.92 | 7.20 |

**Figure 3S.** Cole-Cole plots of FFT impedance spectra of the DOPC:Chol (molar % ratio 6:4) vesicle fusion process on silanized aluminum surface. The applied potential: -0.7 V vs Ag/AgCl/NaCl_(sat.)_. The solid lines represent fits to the data

**Table 2S.** EIS fitting parameters for the data shown in figures 3S

| Vesicle fusion process | R_s_, Ω·cm^2^ | CPE, (µF cm^−2^ s^(α−1)^) | α_CPE_ | R_m_, kΩ·cm^2^ | χ^2^(×10^3^) |
| --- | --- | --- | --- | --- | --- |
| 5 s | 29.71 ± 0.29 | 1.94 ± 0.08 | 0.92 | 76.25 ± 72.07 | 1.65 |
| 140 s | 29.39 ± 0.29 | 1.86 ± 0.07 | 0.92 | 56.87 ± 61.69 | 1.66 |
| 215 s | 29.33 ± 0.29 | 1.81 ± 0.07 | 0.92 | 55.45 ± 56.54 | 1.56 |
| 280 s | 29.17 ± 0.29 | 1.72 ± 0.06 | 0.92 | 50.51 ± 44.67 | 1.56 |
| 350 s | 29.02 ± 0.29 | 1.62 ± 0.06 | 0.92 | 53.03 ± 47.01 | 1.60 |
| 425 s | 28.78 ± 0.28 | 1.55 ± 0.05 | 0.92 | 59.86 ± 53.37 | 1.42 |
| 500 s | 28.6 ± 0.28 | 1.44 ± 0.05 | 0.92 | 37.64 ± 19.64 | 1.40 |
| 565 s | 28.37 ± 0.26 | 1.36 ± 0.04 | 0.92 | 36.77 ± 16.64 | 1.23 |
| 915-2400 s | 28.39 ± 0.30 | 1.25 ± 0.04 | 0.93 | 43.80 ± 25.52 | 1.64 |

**Figure 4S.** Cole-Cole plots of electrochemical impedance spectroscopy of the OTS SAM regeneration for DOPC:Chol (molar % ratio 6:4) hybrid bilayer formation on the metallurgical aluminum surface. The applied potential for EI measurements: -0.7 V vs Ag/AgCl/NaCl_(sat.)_. The solid lines represent fits to the data

**Table 3S.** EIS fitting parameters for the data shown in figures 4S

| Surface | R_s_, Ω·cm^2^ | CPE, (µF cm^−2^ s^(α−1)^) | α_CPE_ | R_m_, kΩ·cm^2^ | χ^2^(×10^5^) |
| --- | --- | --- | --- | --- | --- |
| SAM before 1st hBLM formation | 38.98 ± 0.27 | 2.38 ± 0.03 | 0.93 | — | 10.00 |
| SAM before 2nd hBLM formation | 39.61 ± 0.39 | 3.26 ± 0.06 | 0.91 | — | 20.15 |
| SAM before 6th hBLM formation | 41.63 ± 0.47 | 3.64 ± 0.09 | 0.89 | — | 27.83 |
| hBLM formation 1st time | 44.5 ± 0.16 | 1.21 ± 0.01 | 0.95 | 62.76 ± 8.51 | 3.25 |
| hBLM formation 3rd time | 45.06 ± 0.27 | 1.34 ± 0.02 | 0.94 | 40.29 ± 7.38 | 8.19 |
| hBLM formation 7th time | 45.08 ± 0.26 | 1.54 ± 0.02 | 0.93 | 34.35 ± 5.61 | 7.29 |

**Figure 5S.** Electrochemical impedance spectra in Cole-Cole plot of melittin interaction: **a** and **c** – with DOPC:Chol (molar % ratio 6:4), **b** and **d** – with DOPC hybrid bilayer lipid membranes. The applied potential: -0.7 V vs Ag/AgCl/NaCl_(sat.)._ The solid lines represent fits to the data

**Table 4S.** EIS fitting parameters for the data shown in figures 5S

| Surface | R_s_, Ω·cm^2^ | CPE, (µF cm^−2^ s^(α−1)^) | α_CPE_ | R_m_, kΩ·cm^2^ | χ^2^(×10^5^) |
| --- | --- | --- | --- | --- | --- |
| 100 nM melittin interaction with nM DOPC | | | | | |
| DOPC | 32.27 ± 0.081 | 1.54 ± 0.01 | 0.94 | 79.093 ± 21.29 | 15.48 |
| after interaction | 32.9 ± 0.058 | 1.78 ± 0.01 | 0.93 | 35.55 ± 3.44 | 7.52 |
| 200 nM melittin interaction with DOPC:Chol (molar % ratio 6:4) | | | | | |
| DOPC:Chol (molar % ratio 6:4) | 34.59 ± 0.14 | 1.38 ± 0.02 | 0.93 | 23.76 ± 2.60 | 35.77 |
| after interaction | 34.54 ± 0.11 | 1.47 ± 0.02 | 0.93 | 17.01 ± 1.03 | 18.75 |
| 400 nM melittin interaction DOPC | | | | | |
| DOPC | 46.04 ± 0.13 | 1.742 ± 0.02 | 0.92 | 12.74 ± 0.69 | 18.97 |
| after interaction | 33.79 ± 0.38 | 3.53 ± 0.12 | 0.89 | 8.242 ± 1.15 | 70.70 |
| 400 nM melittin interaction DOPC:Chol (molar % ratio 6:4) | | | | | |
| DOPC:Chol (molar % ratio 6:4) | 34.92 ± 0.085 | 1.22 ± 0.01 | 0.94 | 24.45 ± 1.50 | 12.72 |
| after interaction | 34.19 ± 0.15 | 2.60 ± 0.05 | 0.90 | 8.95 ± 0.64 | 36.32 |

References

1. Xiaojun Han, Kevin Critchley, Lixin Zhang, Singh N. D. Pradeep, Richard J. Bushby, Stephen D. Evans (2006) A Novel Method To Fabricate Patterned Bilayer Lipid Membranes. Langmuir 23: 1354-1358. [↑](#endnote-ref-1)
